# Supplementary material for: Oncogenic Targets Regulated by Tumor-Suppressive miR-30c-1-3p and miR-30c-2-3p: TRIP13 Facilitates Cancer Cell Aggressiveness in Breast Cancer
Source: Cancers (Basel). 2023 Aug 21;15(16):4189. doi: 10.3390/cancers15164189 (PMC10453418; doi:10.3390/cancers15164189)
Supplement: Supplementary file 1 [file cancers-15-04189-s001.zip › cancers-2493032-supplementary-Figures Cancers, Oncogenic targets regulated by miR-30c-3p in breast cancer (July 26, 2023 re-revise version).pptx]

## Slide 1
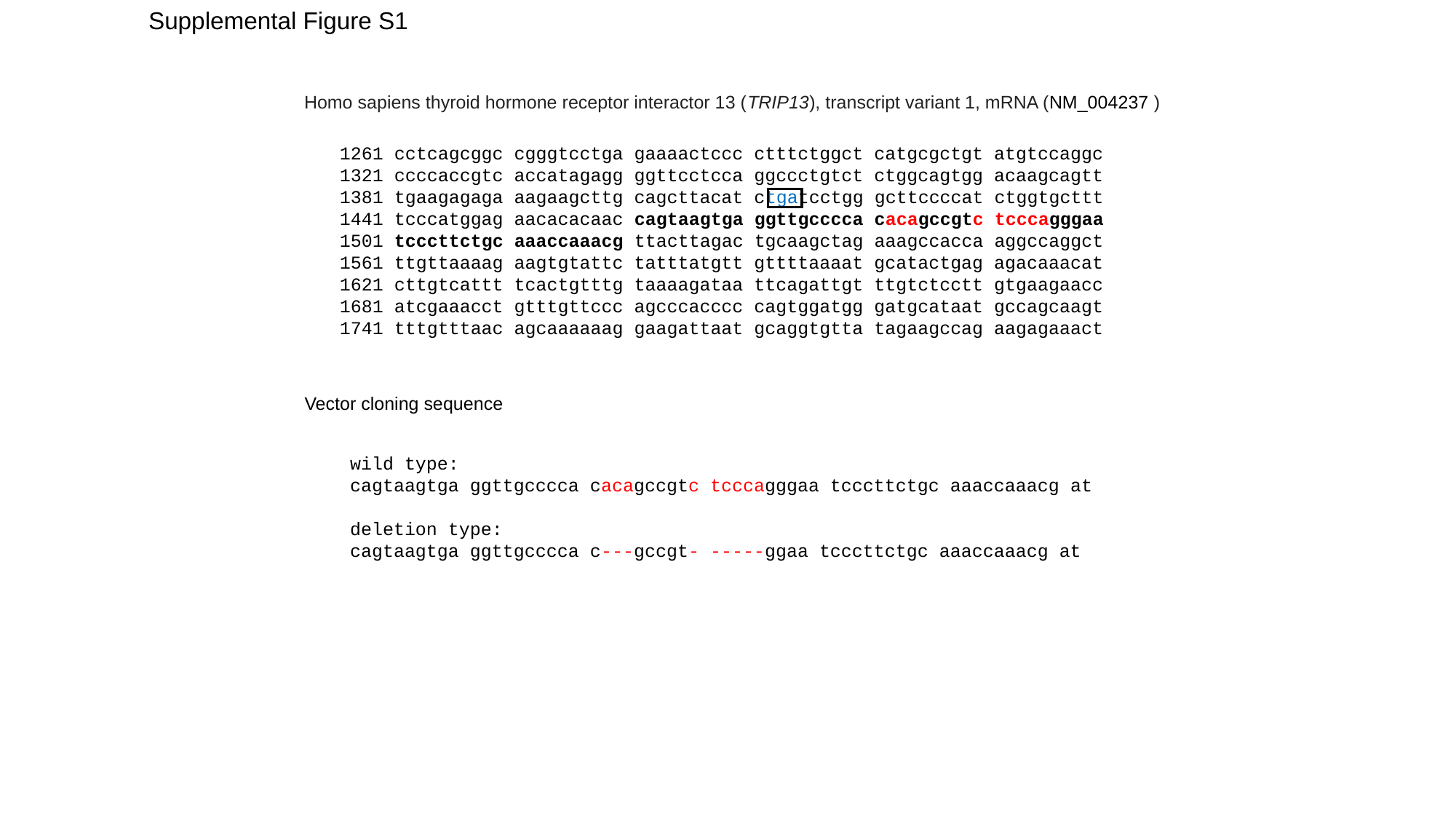

Supplemental Figure S1
Homo sapiens thyroid hormone receptor interactor 13 (TRIP13), transcript variant 1, mRNA (NM_004237 )
 1261 cctcagcggc cgggtcctga gaaaactccc ctttctggct catgcgctgt atgtccaggc
 1321 ccccaccgtc accatagagg ggttcctcca ggccctgtct ctggcagtgg acaagcagtt
 1381 tgaagagaga aagaagcttg cagcttacat ctgatcctgg gcttccccat ctggtgcttt
 1441 tcccatggag aacacacaac cagtaagtga ggttgcccca cacagccgtc tcccagggaa
 1501 tcccttctgc aaaccaaacg ttacttagac tgcaagctag aaagccacca aggccaggct
 1561 ttgttaaaag aagtgtattc tatttatgtt gttttaaaat gcatactgag agacaaacat
 1621 cttgtcattt tcactgtttg taaaagataa ttcagattgt ttgtctcctt gtgaagaacc
 1681 atcgaaacct gtttgttccc agcccacccc cagtggatgg gatgcataat gccagcaagt
 1741 tttgtttaac agcaaaaaag gaagattaat gcaggtgtta tagaagccag aagagaaact
Vector cloning sequence
wild type:
cagtaagtga ggttgcccca cacagccgtc tcccagggaa tcccttctgc aaaccaaacg at
deletion type:
cagtaagtga ggttgcccca c---gccgt- -----ggaa tcccttctgc aaaccaaacg at

## Slide 2
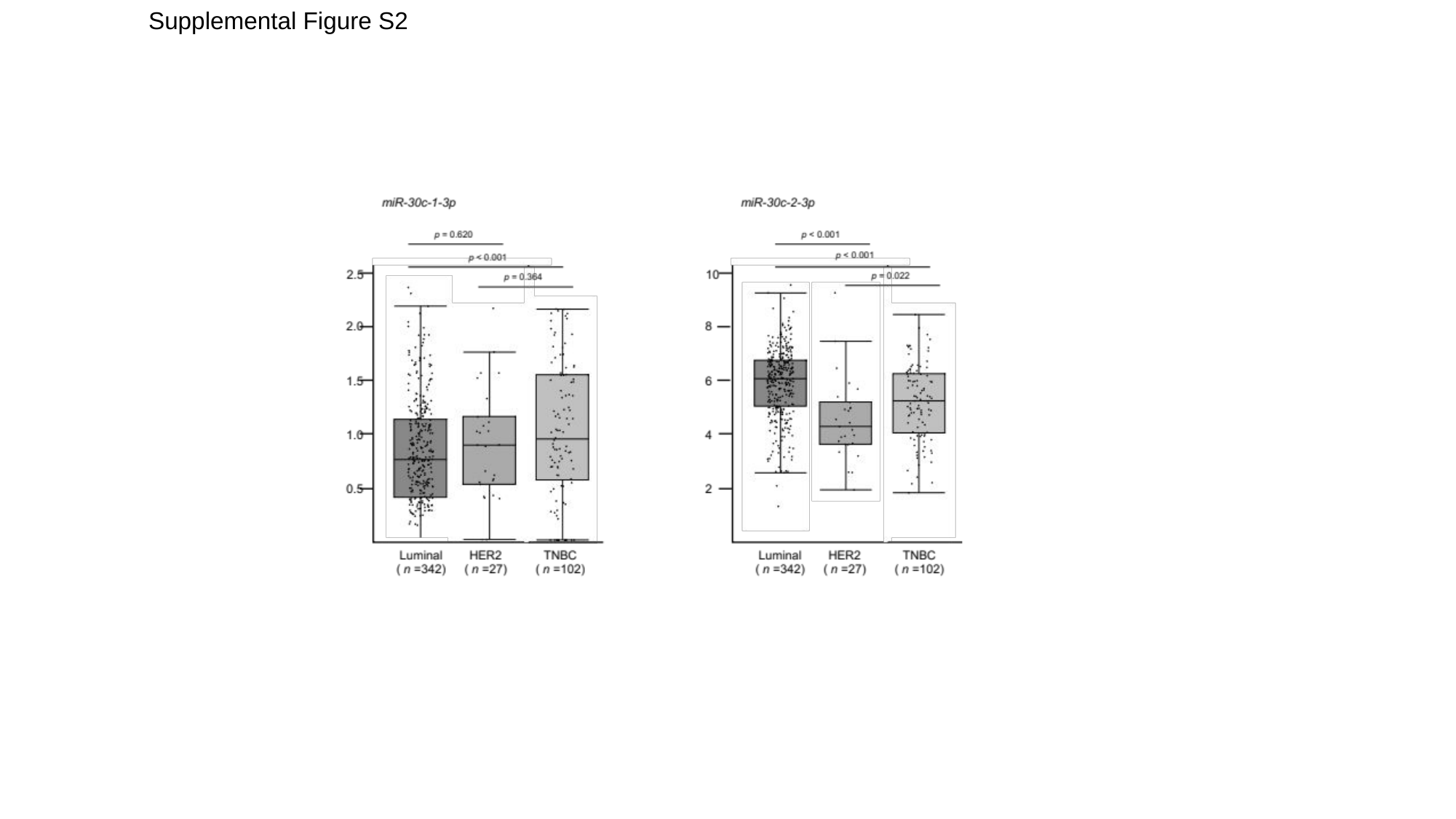

Supplemental Figure S2

## Slide 3
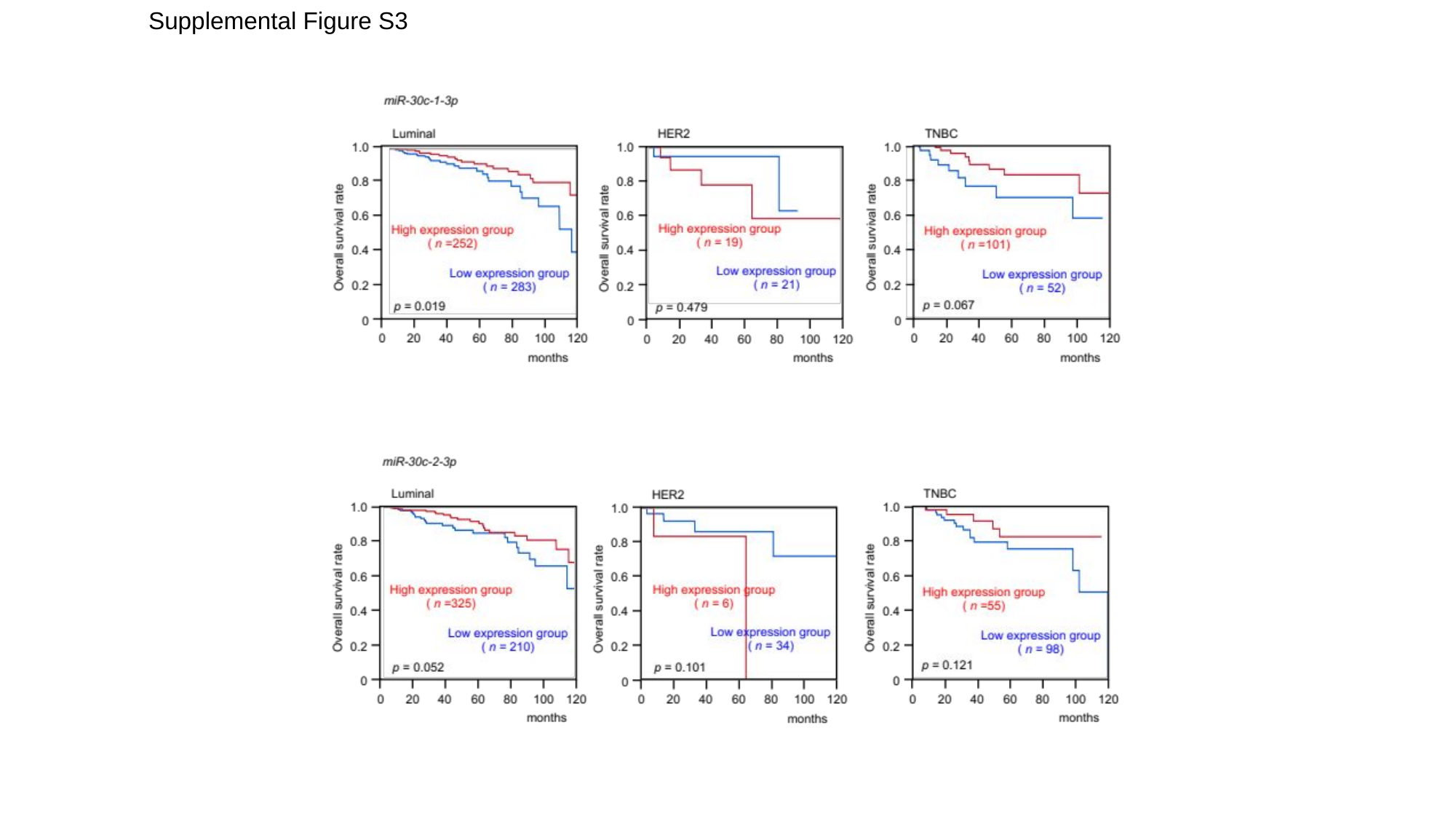

Supplemental Figure S3

## Slide 4
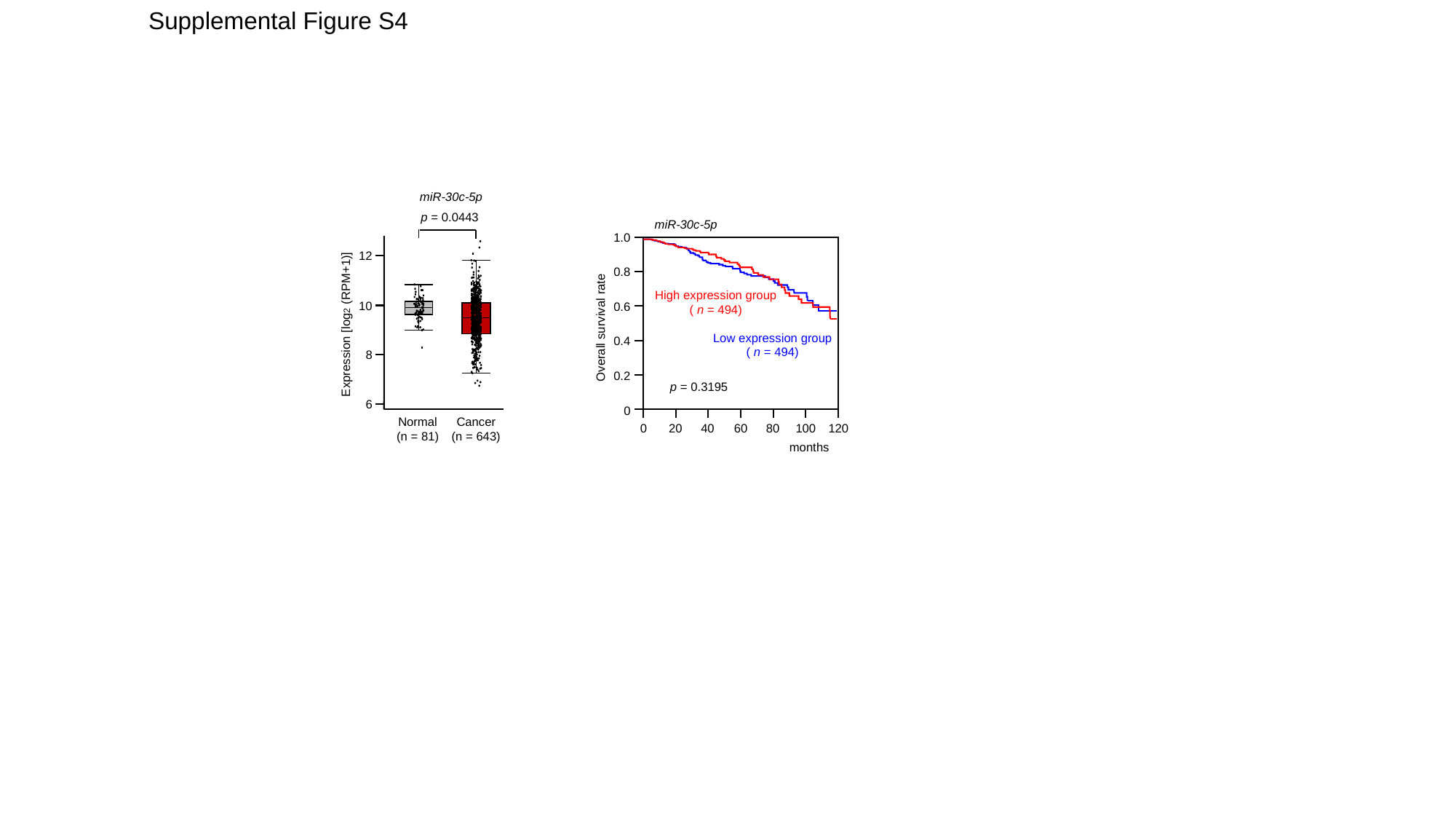

Supplemental Figure S4
miR-30c-5p
p = 0.0443
12
10
Expression [log2 (RPM+1)]
8
6
Normal
(n = 81)
Cancer
(n = 643)
miR-30c-5p
1.0
0.8
High expression group
( n = 494)
0.6
Overall survival rate
Low expression group
( n = 494)
0.4
0.2
0
0
20
40
60
80
100
120
months
p = 0.3195

## Slide 5
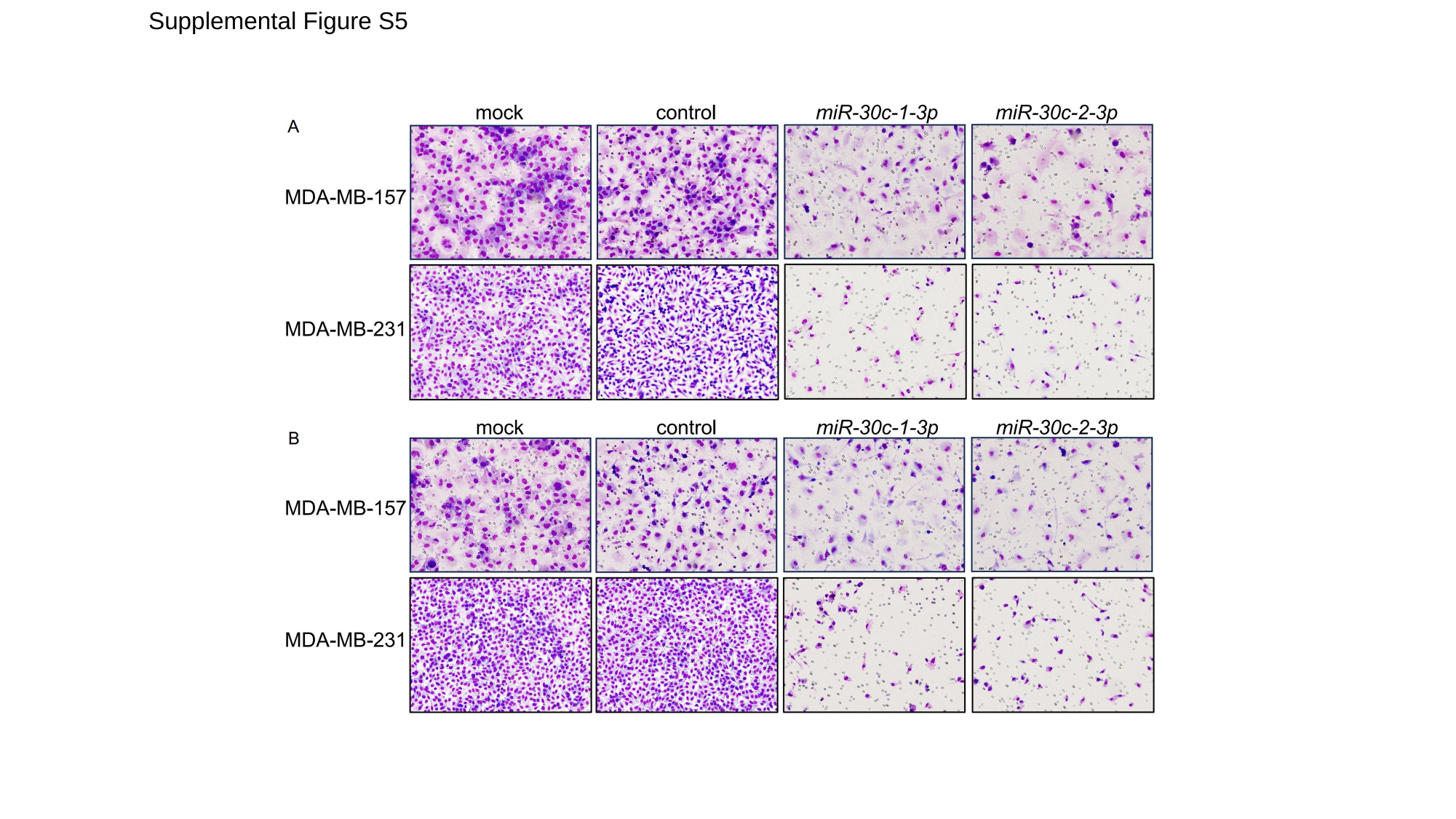

Supplemental Figure S5

## Slide 6
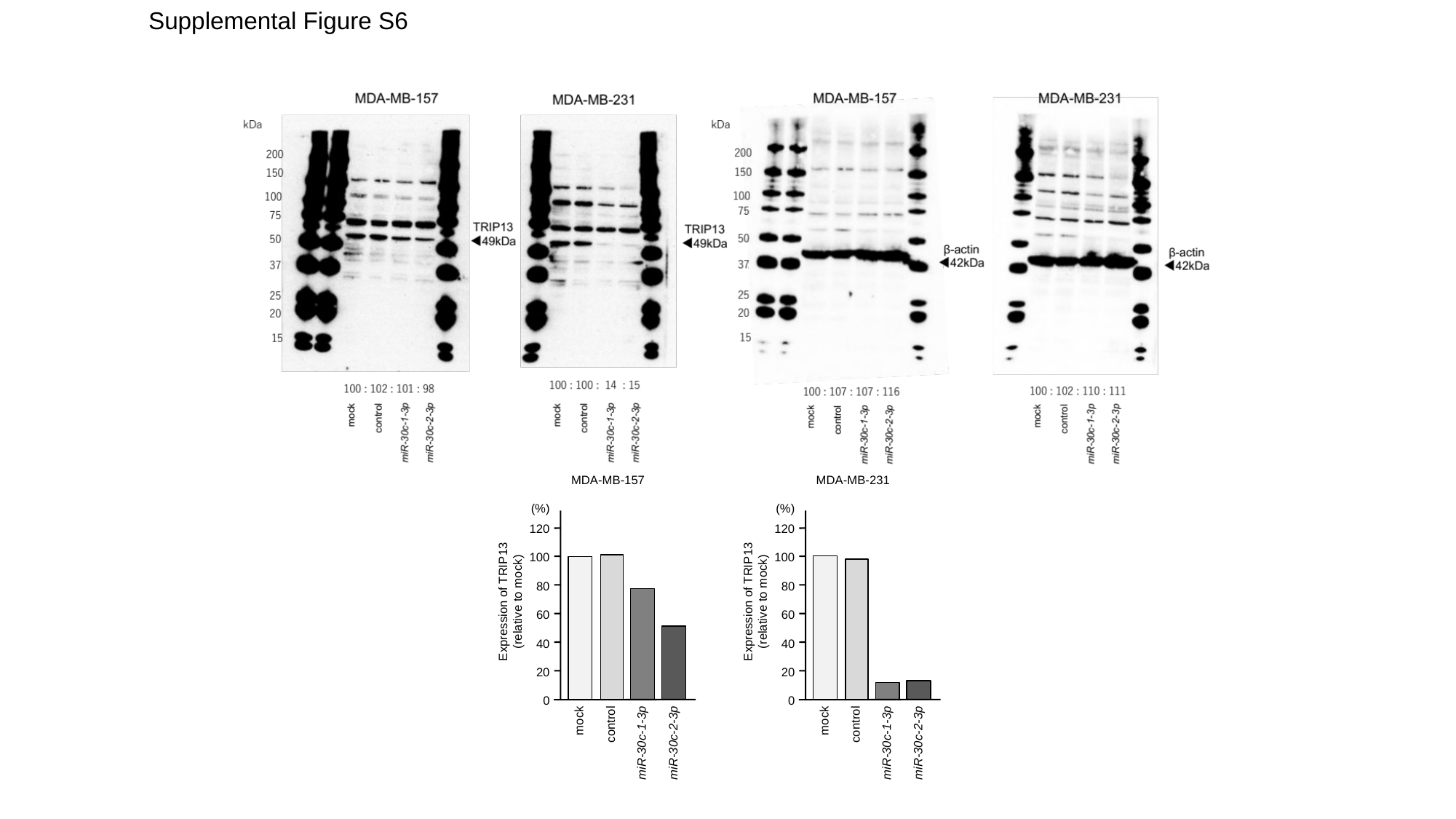

Supplemental Figure S6
MDA-MB-157
(%)
120
100
80
Expression of TRIP13
(relative to mock)
60
40
20
0
mock
control
miR-30c-1-3p
miR-30c-2-3p
MDA-MB-231
(%)
120
100
80
Expression of TRIP13
(relative to mock)
60
40
20
0
mock
control
miR-30c-1-3p
miR-30c-2-3p

## Slide 7
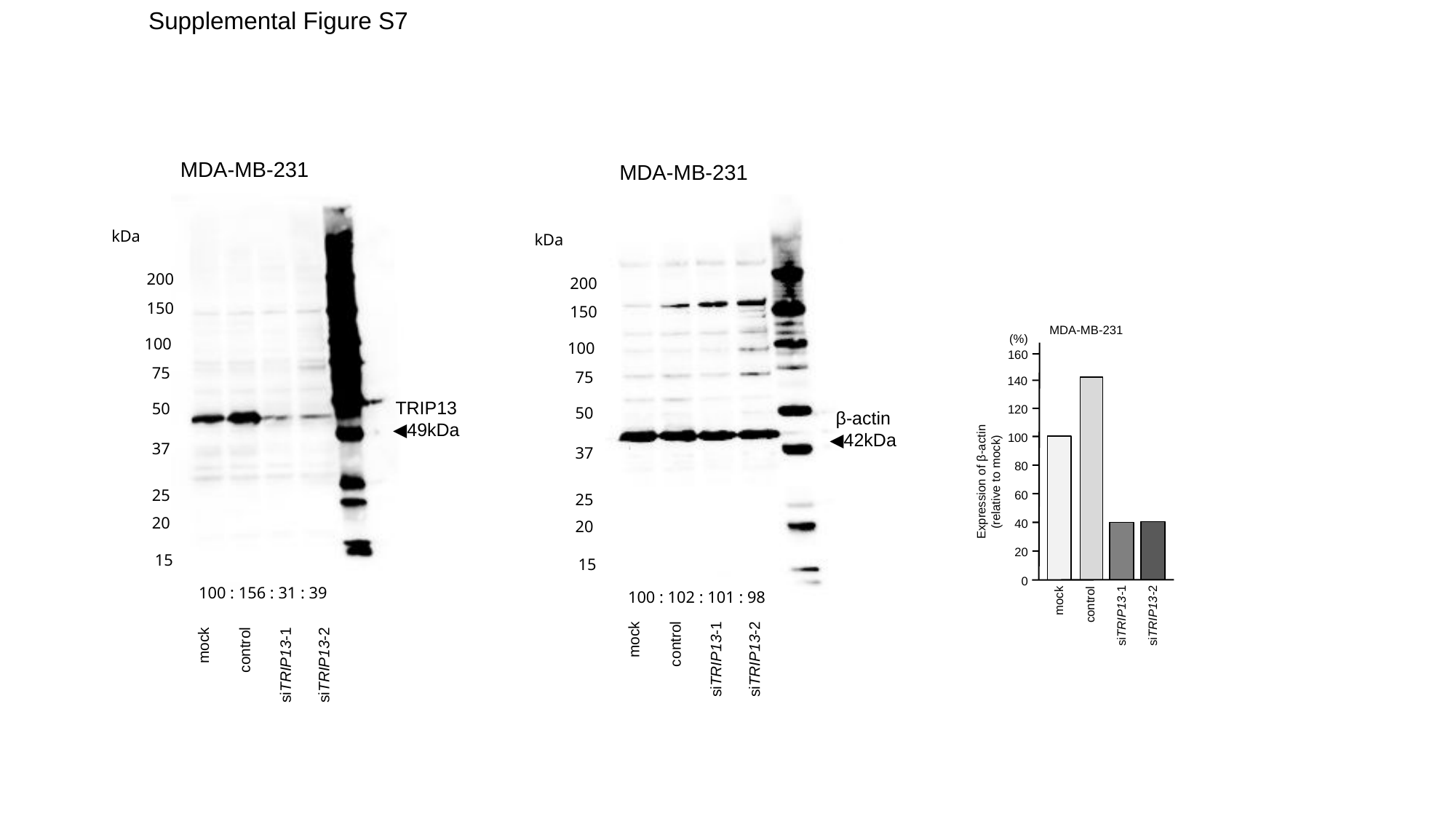

Supplemental Figure S7
MDA-MB-231
MDA-MB-231
kDa
kDa
200
150
100
75
50
37
25
20
15
200
150
100
75
50
37
25
20
15
TRIP13
◀49kDa
β-actin
◀42kDa
100 : 156 : 31 : 39
100 : 102 : 101 : 98
mock
control
siTRIP13-1
siTRIP13-2
mock
control
siTRIP13-1
siTRIP13-2
MDA-MB-231
(%)
120
100
80
Expression of β-actin
(relative to mock)
60
40
20
0
mock
control
siTRIP13-1
siTRIP13-2
160
140
